# Robotic coronary revascularization in Europe, state of art and future of EACTS-endorsed Robotic Cardiothoracic Surgery Taskforce

**DOI:** 10.1093/icvts/ivac108

**Published:** 2022-05-18

**Authors:** Matto Pettinari, Monica Gianoli, Meindert Palmen, Stepan Cerny, Burak Onan, Sandeep Singh, Patrique Segers, Cengiz Bolcal, Cem Alhan, Emiliano Navarra, Herbert De Praetere, Jan Vojacek, Theodor Cebotaru, Paul Modi, Fabien Doguet, Ulrich Franke, Ahmed Ouda, Ludovic Melly, Ghislain Malapert, Louis Labrousse, Alfonso Agnino, Tine Philipsen, Jean-Luc Jansens, Thierry Folliguet, Daniel Pereda, Francesco Musumeci, Piotr Suwalski, Koen Cathenis, Frank Van Praet, Johannes Bonatti, Wouter Oosterlinck, the European Robotic CardioThoracic Surgeons (ERCTS), Stepan Cerny, Wouter Oosterlinck, Burak Onan, Sandeep Singh, Patrique Segers, Cengiz Bolcal, Cem Alhan, Emiliano Navarra, Matteo Pettinari, Frank Van Praet, Herbert De Praetere, Jan Vojacek, Theodor Cebotaru, Paul Modi, Fabien Doguet, Ulrich Franke, Ahmed Ouda, Ludovic Melly, Ghislain Malapert, Louis Labrousse, Monica Gianoli, Alfonso Agnino, Tine Philipsen, Jean-Luc Jansens, Thierry Folliguet, Meindert Palmen, Daniel Pereda, Francesco Musumeci, Piotr Suwalski, Koen Cathenis, Johannes Bonatti

**Affiliations:** 1 Department of Cardiac Surgery, Ziekenhuis Oost Limburg, Genk, Belgium; 2 Department of Cardiac Surgery, University Medical Centre Utrecht, Utrecht, Netherlands; 3 Department of Cardiac Surgery, Leiden University Medical Center, Leiden, Netherlands; 4 Department of Cardiac Surgery, Na Homolce Hospital, Prague, Czech Republic; 5 Department of Cardiac Surgery, Istanbul Mehmet Akif Ersoy Cardiovascular Surgery Hospital, University of Health Sciences, Istanbul, Turkey; 6 Department of Cardiac Surgery, ISALA Hospital, Zwolle, Netherlands; 7 Department of Cardiac Surgery, Maastricht University Medical Center, Maastricht, Netherlands; 8 Department of Cardiac Surgery, Gulhane Education ve Research Hospital, Ankara, Turkey; 9 Department of Cardiac Surgery, Acibadem Maslak Hospital, Acibadem University, Istanbul, Turkey; 10 Department of Cardiac Surgery, Cliniques Univesitaires Saint Luc, Brussels, Belgium; 11 Department of Cardiac Surgery, Imelda Hospital Bonheiden, Bonheiden, Belgium; 12 Department of Cardiac Surgery, University Hospital Hradec Kralove, Hradec Kralove, Czech Republic; 13 Department of Cardiac Surgery, MONZA Hospital, Bucharest, Romania; 14 Department of Cardiac Surgery, Liverpool Heart and Chest, Liverpool, United Kingdom; 15 Department of Cardiac Surgery, Private Hospital Jacques Cartier, Massy, France; 16 Department of Cardiac Surgery, Robert Bosch Hospital, Stuttgart, Germany; 17 Department of Cardiac Surgery, University Hospital Zurich, Zurich, Switzerland; 18 Department of Cardiac Surgery, CHU UCL Namur—Site Godinne, Namur, Belgium; 19 Department of Cardiac Surgery, CHU Dijon, Dijon, France; 20 Department of Cardiac Surgery, University Hospital Bordeaux, Bordeaux, France; 21 Department of Cardiac Surgery, Humanitas Gavazzeni, Bergamo, Italy; 22 Department of Cardiac Surgery, University Hospital Ghent, Ghent, Belgium; 23 Department of Cardiac Surgery, Erasme Hospital Brussels, Brussels, Belgium; 24 Department of Cardiac Surgery, Hôpital Henri MONDOR, Assistance Publique-Hôpitaux de Paris, Université Paris 12, Créteil, France; 25 Department of Cardiac Surgery, Hospital Clínic de Barcelona, Barcelona, Spain; 26 Department of Cardiac Surgery, San Camillo Hospital, Rome, Italy; 27 Department of Cardiac Surgery, Central Teaching Hospital of the Ministry of the Interior and Administration, Centre of Postgraduate Medical Education, Warsaw, Poland; 28 Department of Cardiac Surgery, AZ Maria Middelares, Ghent, Belgium; 29 Department of Cardiac Surgery, Cardiovascular Center, OLV Clinic, Aalst, Belgium; 30 Department of Cardiac Surgery, University of Pittsburgh Medical Center (UPMC), Pittsburgh, PA, USA; 31 Department of Cardiovascular Sciences, University Hospital Leuven, KU Leuven, Leuven, Belgium

## BACKGROUND

It has been >20 years ago that robotic-assisted coronary artery bypass grafting (RA-CABG) has been introduced, but the adoption of this technique is still rather limited worldwide, although recently a slight increase in numbers has been documented in Europe [1, 2]. Like many novelties, after the introduction, it has been picked up by only a few dedicated surgeons in highly specialized centres. Due to limited series, based mostly on single-centre experiences, extensive clinical outcome data and results on long-term benefits are lacking as well as the acknowledgement in international cardiosurgical society and anchorage in EACTS-supported guidelines. The limited number of robotic platforms and high procedural costs combined with the absence of dedicated training programs are considered to be responsible for reduced adoption. The safety of robotic techniques, the benefit of the left internal mammary artery (LIMA) to left anterior descending (LAD) over percutaneous coronary interventions (PCI) and hybrid procedures have also been questioned. Nonetheless, after 20 years, the robotic surgical technique has evolved. Consequentially, the number of off-pump robot-assisted minimally invasive direct coronary artery bypass (RA-MIDCAB) has rapidly grown lately [1, 2]. It seems that the robotic approach to ischaemic heart disease has earned its place in our surgical armamentarium. This editorial will address the current standards of care and future perspectives of robotics in coronary revascularization.

## ROBOTIC-ASSISTED CORONARY REVASCULARIZATION APPROACHES

**Figure 1:**
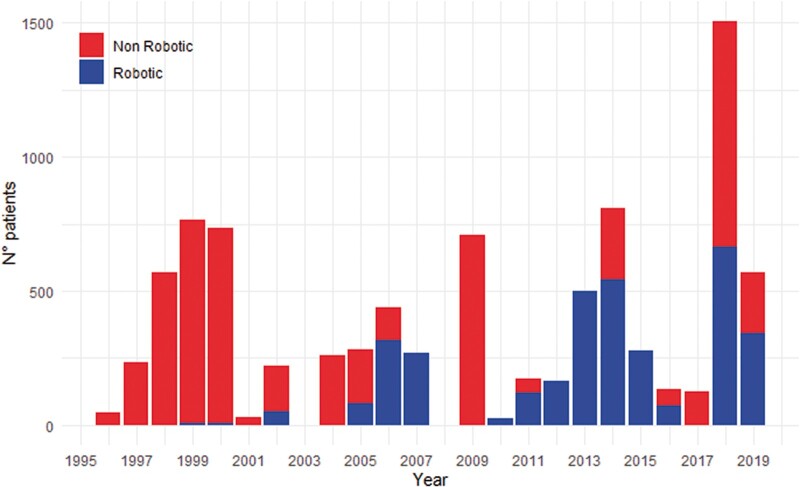
Number of patients treated by minimally invasive coronary artery bypass derived from the published literature available.

The first robotic-assisted coronary revascularization was described in 1999 by Loulmet. In 6 patients, the left internal mammary artery (IMA) was harvested using a robotic approach and subsequently grafted to the LAD coronary artery. In 2 patients, the procedure was performed completely endoscopically. Recently, we witnessed a growing interest in minimally invasive coronary artery bypass grafting (CABG), performed not only robotically assisted but also under ‘direct view’ or videoscopy assisted. Nevertheless, non-robotic procedures showed mostly inferior outcomes when compared to the robotic ones, in terms of major acute cardiac and cerebrovascular events (MACCE), duration of intensive care unit stay and postoperative pain. In a recent study, Bonatti *et al.* [3] reviewed the 25-year-long journey of minimally invasive coronary surgery, demonstrating how robotic activity increased after the FDA approval of the Da Vinci system (Intuitive Surgical, Sunnyvale, CA, USA) in 2000. Afterward, fluctuation in the number of performed procedures showed a first peak in 2006 and the second one in 2014 (Fig. 1). In Europe, where robotic surgery did not reach the popularity achieved in the USA, probably due to the differences in the economical asset of the Public Health System, we recently witnessed a doubling in the numbers of centres performing robotic coronary revascularization between 2016 and 2019. Maintaining the same rate of growth, we expect that the European robotic CABG volume could equal the US volume in the next 5 years [2].

Essentially, 2 different coronary revascularization procedures can be performed using the robotic platform: RA-MIDCAB and totally endoscopic coronary artery bypass graft (TECAB). In RA-MIDCAB, surgeons use the robotic system to harvest 1 or 2 IMA’s, open the pericardium and identify coronary targets. Through a small left anterior thoracotomy, coronary anastomosis is manually crafted in an off-pump setting. The second procedure, TECAB, is completely robotically performed, and therefore technically challenging for the surgeons. Without additional thoracotomy, target vessel stabilization and grafting are completely performed endoscopically. TECAB in its beating heart version is only feasible using a robotic endostabilizer. Also, robotic suturing of the anastomosis is challenging and automatic connector devices have been developed. Unfortunately, both technologies due to the lack of demand have been on hold which prevents the further spread of this procedure. A few centres still perform it using work around but RA-MIDCAB is currently the most adopted technique.

## COMPARISON TO CONVENTIONAL CABG

**Figure 2:**
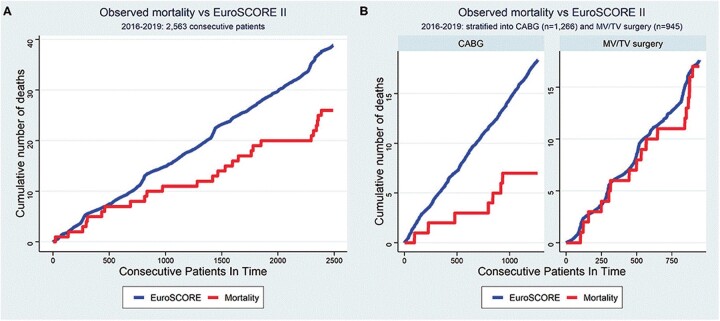
Expected versuss observed mortality after coronary and robotic procedure. Taken from Cerny *et al.* [2].

Since Loulmet’s first report, clinical outcomes after robotic CABG were obtained mostly from single-centre retrospective observational data. Several series showed excellent results with a low incidence of mortality, stroke and myocardial infarction. Robotic techniques also showed a reduction in pneumonia, postoperative pain, transfusion requirement and recovery time when compared to conventional CABG. Bonatti’s review [3] on 11 135 patients reported hospital mortality of 1% and a stroke rate of 0.6%. The revision rate for bleeding was 2.5% and a renal failure rate of 0.9% was noted. Wound infections occurred at a rate of 1.2% and postoperative hospital stay was close to 5 days. An average of 1.3 grafts were performed in <4 h of operative time adopting 6 main versions of minimal access and robotically assisted CABG. The review concluded that less invasive and robotically assisted versions of coronary bypass grafting are carried out with an adequate safety level while surgical trauma is significantly reduced when compared to standard CABG. Also, current European outcomes for robotic CABG, on 1266 patients, are comparatively very encouraging with very low mortality (0.6%) and no strokes (Fig. 2). Revision for bleeding rate of 2.1% is acceptable and the low (2.6%) conversion rate likely reflects a learning curve of the robotic cardiac surgery community and demonstrates that the procedures have become more standardized [2]. A further recent meta-analysis comparing TECAB and RA-MIDCAB to conventional CABG, demonstrated a reduction at 1 year of the composite outcome of death, myocardial infarction and stroke in favour of the robotic procedures. Also, outcomes such as graft patency and the need for repeat revascularization (RR) were excellent. In literature, a similar rate of RR for the 2 procedures is reported, demonstrating that robotic CABG meets the standards of open CABG concerning graft quality. Most of the RA-MIDCAB or TECAB procedures were performed for single-vessel disease; however, experienced teams demonstrated the feasibility of performing multiple arterial bypass using both IMAs, with an average of 2.4 anastomosis/patient, in multivessel disease [4]. In Balkhy series, the right internal mammary artery (RIMA) was used as an *in situ* graft in 124 cases (84%) and as a free T-graft in 24 cases (16%) cases. The use of bilateral mammary artery increased from 23% in the first 5 years to 53% in the last 2 years. Also, for these complex procedures, perioperative mortality and morbidity were low. Mortality was 0.7%, myocardial infarction 0.3–1.1% and stroke 0.5%. Length of hospital stay was quite short reporting an average of 3 days. The authors concluded that robotic TECAB allows the routine harvesting and use of the RIMA graft in a safe and reproducible manner. In the last years, besides the implementation of surgical strategy with the adoption of completely arterial revascularization for the left coronaries, the complexity of the patient referred to robotic revascularization increased. Obesity, elderly, redo operation or chronic pulmonary diseases in the past considered as a contra-indication for MIDCAB and TECAB became lately more common characteristics among the robotic population [4]. In fact, despite an intrinsic increased operative risk, those patients are the most advantaged by a sternal sparing approach and an early recovery.

## THE BENEFIT OF THE LEFT INTERNAL MAMMARY ARTERY–LEFT ANTERIOR DESCENDING OVER PERCUTANEOUS CORONARY INTERVENTIONS

**Figure 3:**
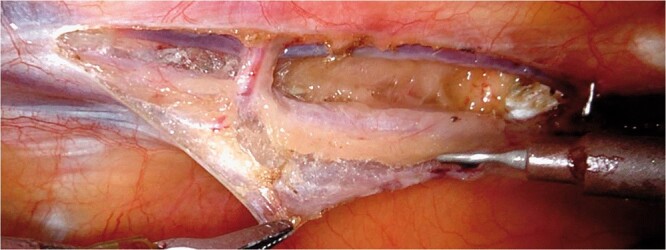
Intraoperative view of left mammary artery harvesting.

CABG and PCI are well-established revascularization strategies for proximal LAD lesions, both are considered as the first option in the European guidelines for revascularization. However, even after 2 decades, minimally invasive surgical revascularization has never been included in the general recommendations. Surgical revascularization (LITA to LAD) offers a better long-term survival and decreased demand for RR, while PCI offers a less-invasive nature of the treatment. PCI represents a valuable alternative for old and multimorbid patients with high risk for surgery or simply a temporary solution to delay surgery in young and still fit patients. In the past, the SIMA trial showed the superiority of the mammary artery when compared to the bare-metal stent in terms of RR up to 10 years. Lately, the introduction of drug-eluting stents (DES) has changed the equation somewhat during the last decade. Although DES reduced the incidence of early restenosis, its inferiority compared to CABG was demonstrated in several meta-analyses [5]. Outcomes in these studies were congruent: mortality and MACCE were similar in both groups, while the need for RR was higher using DES. The second generation of DES reduced the need for RR, but even when PCI was performed FFR guided, targeting only the functionally significant lesions and avoiding unnecessary stenting and herewith stent-related complications, the occurrence of MACCE within 1 year was higher in the PCI group when compared to CABG [6]. For isolated LAD lesions, minimally invasive surgical revascularization with IMA to LAD showed lower RR, and higher freedom from angina especially when a longer stent (>30 mm) was deemed necessary with percutaneous revascularization. Similar findings were described for left main disease, by a recent meta-analysis, demonstrating lower rates of late target vessel RR in patients undergoing MIDCAB when compared to PCI [7]. In experienced robotic teams, bilateral IMA harvesting and robot-assisted target vessel revascularization of the left-sided coronary lesions could further improve outcomes and revascularization options. In addition, skeletonization and sternal sparing allow the RIMA to reach various coronary targets [4]. In this setting, the patients receive the advantages of completely arterial revascularization with the benefit of a less-invasive approach. Robotically assisted placement of bilateral IMAs and combination with PCI in advanced hybrid coronary revascularization for the complex multivessel disease has also been successfully carried out. In fact, the use of mammary arteries for surgical revascularization may have specific advantages when compared to PCI, which can be attributed to their specific anatomical and biological characteristics. IMAs produce a high level of nitric oxide inducing endothelial-dependent vasodilation effect also in the grafted coronaries and providing a ‘surgical collateralization’, prolonging life by preventing myocardial infarction [8]. Although most of these considerations indicate the need for surgical revascularization of, at least the more complex, LAD lesions, inappropriate or traumatic IMA graft harvesting techniques could easily impair graft patency and therefore outcome [9]. Nowadays, robotic-assisted harvesting of the ITAs can be performed with minimal tissue damage (Fig. 3), resulting in optimal graft patency while reducing complications like (sternal) wound infections [3, 4]. Furthermore, a more extensive intraoperative graft quality control using a Transit Time Flowmeter, highly recommended during minimally invasive CABG, permits direct analysis of the final results with the aim to improve early and late graft patency.

## HYBRID REVASCULARIZATION

Hybrid coronary revascularization (HCR) combines surgical coronary revascularization (LIMA-to-LAD graft) with percutaneous coronary revascularization (PCI of significantly affected non-LAD lesions). Robotic-assisted techniques enabling LITA-to-LAD grafting provide the patient with the survival benefit of the LITA–LAD grafting while avoiding the risks of cardiopulmonary bypass, aortic manipulations and sternotomy. Furthermore, integrated PCIs provide the patient with the least invasive HCR option, achieving complete revascularization of all diseased coronary arteries. The use of the second-generation DES is of paramount importance in the treatment of non-LAD coronary pathology and provides a valuable alternative to surgical revascularization of non-LAD targets using a venous graft, the latter being notorious for future atherosclerotic degeneration resulting in high short- and long-term failure rates [5–7].

Several single-centre studies comparing HCR to CABG have been published so far. Improvements in short-term outcomes in terms of hospital stay and transfusion requirements have been described in favour of HCR. Long-term data demonstrated at nearly 10-year follow-up similar outcomes in terms of composite end-point of death, RR and new myocardial infarction. Clear data comparing HCR and total arterial open CABG are still lacking in the literature and there is definitely a need for prospective randomized comparisons.

In conclusion, the ideal candidates for (robotically-assisted) HCR could be patients with multivessel disease with a complex LAD lesion suitable for LIMA–LAD grafting, associated with non-complex non-LAD lesions (SYNTAX score <22) suitable for PCI. Importantly, HCR should not be considered as an alternative to CABG for patients with diffuse complex coronary pathology (SYNTAX >22) but should be viewed as an alternative to multivessel PCI in patients with LAD disease having low-intermediate SYNTAX score. The more complex disease may be amenable to advanced hybrid revascularization concepts including robotic double IMA grafting for the left coronaries and PCI for the right side. Nevertheless, each patients’ specific decision needs to be discussed by the heart team to define the most appropriate tailored approach.

## TRAINING AND QUALITY CONTROL

RA-CABG represents roughly 1–3% of total CABG procedures performed in Europe [1]. Reasons for limited adoption might include high initial investment and high procedural costs of the robotic platform and the demand for a high level of expertise for all teams involved in the procedure. The lack of a formalized training program also plays an important role. In 2016, a joint Society of Thoracic Surgeons and American Association for Thoracic Surgery task force was created to address the gaps in RA-CABG adoption and performance implementation. Optimal surgeon training has been identified as a critical component of procedural development across various domains. The single-centre series evaluated the effect of the level of surgical experience on the efficiency of the procedures. It was shown that between 5 and 20 cases, IMA harvesting time decreased significantly. Similar trends were observed for the time needed for port placement and coronary artery grafting and consequently for the overall operative procedural duration [10]. Surgeons’ learning curve may potentially also affect procedural success. Although the steepness of the learning curve may vary amongst surgeons, it has been described that in experienced teams with more than 50 procedures, a decreased (decrease) in conversion rate, reoperation need and mortality can be observed [10]. Beating heart off-pump surgical revascularization skills and a dedicated team approach, may also shorten this learning curve, allowing for safe implementation and paving the road towards more complex procedures such as multivessel completely arterial revascularization.

Benchmarking RA-CABG outcomes, creating both a nationwide and an international registry, is considered to be a necessary step to guarantee quality control. Apart from benchmarking and quality control, a registry may allow for a large retrospective cohort study comparing RA-CABG with both conventional CABG and multivessel PCIs. Furthermore, we expect that a standard of reference will also improve the performances of the individual robotic centres.

## CONCLUSION

Robotic CABG has been adopted slowly after its initial introduction more than 2 decades ago but gained popularity in the past few years. Being an ideal surgical counterpart for PCI in HCR strategies, we expect that robotic CABG may contribute to a paradigm shift in the treatment of patients with complex multivessel coronary artery disease. Visibility and acceptance of robotic CABG in myocardial revascularization guidelines, the set-up of official international training programmes, procedural benchmarking and active involvement of the international cardiothoracic society are crucial but still lacking to date. The first step towards acknowledgement of the role of robotics in cardiac surgery was taken by the European Society of Thoracic and Cardiovascular Surgery, which supported the implementation of an EACTS-endorsed Robotic Cardiothoracic Surgery Taskforce. The aim of this task force is to analyse actual and future outcomes, promote high-quality team training, stimulate support from the industry and improve the application of future technologies.
